# Regulation of microglia function by neural stem cells

**DOI:** 10.3389/fncel.2023.1130205

**Published:** 2023-03-01

**Authors:** Monique M. A. de Almeida, Kara Goodkey, Anastassia Voronova

**Affiliations:** ^1^Department of Medical Genetics, Faculty of Medicine & Dentistry, Edmonton, AB, Canada; ^2^Faculty of Medicine & Dentistry, Neuroscience and Mental Health Institute, Edmonton, AB, Canada; ^3^Women and Children’s Health Research Institute, 5-083 Edmonton Clinic Health Academy, University of Alberta, Edmonton, AB, Canada; ^4^Department of Cell Biology, Faculty of Medicine & Dentistry, Edmonton, AB, Canada; ^5^Multiple Sclerosis Centre and Department of Cell Biology, Faculty of Medicine & Dentistry, Edmonton, AB, Canada

**Keywords:** NPC, OPC, immunomodulatory, regeneration, multiple sclerosis, remyelination, neuroinflammation, neuroprotection

## Abstract

Neural stem and precursor cells (NPCs) build and regenerate the central nervous system (CNS) by maintaining their pool (self-renewal) and differentiating into neurons, astrocytes, and oligodendrocytes (multipotency) throughout life. This has inspired research into pro-regenerative therapies that utilize transplantation of exogenous NPCs or recruitment of endogenous adult NPCs for CNS regeneration and repair. Recent advances in single-cell RNA sequencing and other “omics” have revealed that NPCs express not just traditional progenitor-related genes, but also genes involved in immune function. Here, we review how NPCs exert immunomodulatory function by regulating the biology of microglia, immune cells that are present in NPC niches and throughout the CNS. We discuss the role of transplanted and endogenous NPCs in regulating microglia fates, such as survival, proliferation, migration, phagocytosis and activation, in the developing, injured and degenerating CNS. We also provide a literature review on NPC-specific mediators that are responsible for modulating microglia biology. Our review highlights the immunomodulatory properties of NPCs and the significance of these findings in the context of designing pro-regenerative therapies for degenerating and diseased CNS.

## Neural stem and precursor cells build and regenerate the brain

Neural stem and progenitor cells (NPCs) were first discovered as radial glia cells lining the embryonic ventricles and were found to express many of classical astrocyte markers, like the glial fibrillary acidic protein (GFAP) (Levitt and Rakic, 1980). These cells were attributed a *bona fide* glial progenitor status, and their primary role was believed to serve as a guide for newborn neuron migration. It was not until late 1990s that radial glia cells were shown to self-renew and were found to have multipotent ability to generate neurons and macroglia (astrocytes and oligodendrocytes), which are properties of stem cells (Malatesta and Götz, 2013). It is now widely accepted that during embryonic and postnatal brain development, NPCs differentiate into neurons, astrocytes and oligodendrocytes in a strict spatio-temporal manner to build the central nervous system (CNS) (Gauthier-Fisher and Miller, 2013; Adnani et al., 2018; Lindsey et al., 2018). Embryonic NPCs also give rise to adult parenchymal OPCs (oligodendrocyte precursor cells), which serve as a reservoir for oligodendrocyte regeneration (Kessaris et al., 2006). Finally, some embryonic NPCs divide more slowly and contribute to the establishment of the adult neurogenic niches (Fuentealba et al., 2015; Furutachi et al., 2015; Yuzwa et al., 2017). In the adult mammalian brain, NPCs are restricted to the subgranular zone (SGZ) of the dentate gyrus in the hippocampus and to the subventricular zone (SVZ) of the lateral ventricles. Adult SVZ and SGZ NPCs remain multipotent throughout life and renew neurons, astrocytes, parenchymal OPCs and oligodendrocytes in a homeostatic and diseased or injured brain (Lagace, 2012; Gage and Temple, 2013; Chaker et al., 2016; Obernier and Alvarez-Buylla, 2019; Xapelli et al., 2020).

## Role of microglia in regulating NPC biology

Many NPC and OPC fates are regulated by neighboring cells in their niche, such as microglia (Miron et al., 2013; Shahriary et al., 2019; Schmidt et al., 2021). Microglia are CNS immune cells that are derived from the embryonic yolk sac progenitors. Microglia migrate into an embryonic brain as early as E (embryonic day) 9 and then proliferate until early postnatal life to populate the murine CNS tissue (Alliot et al., 1999; Ginhoux et al., 2010; Kierdorf et al., 2013). Once the microglia pool is established, microglia survey the environment and become reactive during infection and inflammation (Amici et al., 2017; Shabab et al., 2017). Microglia are constantly surveying the microenvironment and are known to play a role in the resolution of injury and inflammation (Saijo and Glass, 2011; Amici et al., 2017).

Reactive microglia exist in a continuum of states associated with various biological functions, which historically have been categorized into two opposite types: inflammatory (formerly known as M1) and repair or alternate state associated with secretion of anti-inflammatory and pro-regenerative cytokines and chemokines (formerly known as M2). However, it is important to note that there is a continuum of phenotypes between inflammatory and repair states, and microglia can transition between them (Paolicelli et al., 2022). Much of microglia activation and functions (proliferation, survival, migration, and phagocytosis) depends on the factors within the surrounding environment (Rawji and Yong, 2013; Baaklini et al., 2019). In a diseased or injured brain, M1-like microglia secrete pro-inflammatory cytokines, such as IL-1β (interleukin 1 beta), IL-6, TNF-α (tumor necrosis factor alpha) and nitric oxide (Tang and Le, 2016; Guo et al., 2022). While inflammatory microglia play an important role in CNS repair, a sustained and exacerbated response by this subset of microglia has been linked to neurodegeneration and inhibition of CNS regeneration (Tang and Le, 2016; Baaklini et al., 2019; Lloyd et al., 2019; Guo et al., 2022). In agreement, reduction of inflammatory microglia is associated with enhanced CNS regeneration (de Almeida et al., 2023). Repair-associated microglia promote the resolution of inflammation, dead cell and myelin debris phagocytosis and are implicated in the production of pro-regenerative and pro-survival factors (Miron et al., 2013). Enhancement of a repair phenotype in microglia is associated with improved CNS regeneration and repair (Miron et al., 2013; Beier et al., 2017; Chang et al., 2017). Notably, phagocytosis of apoptotic cells is known to promote repair phenotype in microglia and macrophages (Fadok et al., 1998; Arandjelovic and Ravichandran, 2015). However, myelin was shown to induce inflammatory macrophages in a rodent model of a spinal cord injury (SCI) (Kopper et al., 2021). While it is difficult to tease apart the role of dead cells vs. myelin debris phagocytosis with regard to macrophage and microglia polarization, it is known that phagocytosis of myelin debris is essential for efficient CNS remyelination (Plemel et al., 2013; Lampron et al., 2015). In agreement, blocking myelin debris phagocytosis exacerbates neuroinflammation in experimental autoimmune encephalomyelitis (EAE, model of multiple sclerosis), and increasing myelin debris phagocytosis by microglia enhances remyelination in the aging CNS (Grajchen et al., 2020; Rawji et al., 2020).

Thus, in addition to their well-known immune functions such as chemotaxis, phagocytosis, and the secretion of inflammatory cytokines, microglia also play additional vital roles during CNS development, homeostasis and regeneration. During development, microglia exert neuroprotective properties by secreting trophic factors as well as engulfing synapses and live developing NPCs and OPCs (Cunningham et al., 2013; Michell-Robinson et al., 2015; Kaur et al., 2017; Nemes-Baran et al., 2020; Hammond et al., 2021; Rosin et al., 2021; Mehl et al., 2022). In the injured brain, microglia modulate NPC and OPC fates through phagocytosis of progenitors, myelin debris, and secretion of anti- and pro-regenerative cytokines, growth factors and other ligands (Miron et al., 2013; Hayakawa et al., 2016; Li et al., 2017; Dillenburg et al., 2018; Heß et al., 2020; Schmidt et al., 2021; Sherafat et al., 2021).

## “Immune” roles of NPCs in CNS development and regeneration

With the wide adoption of single-cell RNA sequencing and “omics” techniques, it was recently shown that both NPCs and OPCs express genes that are typically attributed to immune cells (Falcão et al., 2018; Kirby et al., 2019; Kirby and Castelo-Branco, 2020; Nicaise et al., 2020; Watson et al., 2020; Belenguer et al., 2021; Willis et al., 2022; Dittmann et al., 2023). For example, NPCs and OPCs express MHC (major histocompatibility complex) class I antigen presentation category genes (Falcão et al., 2018; Kirby et al., 2019; Lin et al., 2021; Dittmann et al., 2023). Subsequently, OPCs were shown to present antigens and activate T-cells in rodent models of multiple sclerosis (Falcão et al., 2018; Kirby et al., 2019; Fernandez-Castaneda et al., 2020). Both NPCs and OPCs can also act as phagocytes. Dcx^+^ (Doublecortin) neuroblasts, which are derived from NPCs and represent committed proliferative progenitors that are destined to differentiate into neurons, phagocytose dead neurons, whereas OPCs can phagocytose myelin debris and prune synapses; properties typically attributed to microglia and macrophages (Lu et al., 2011; Falcão et al., 2018; Auguste et al., 2022). NPCs and OPCs also directly react to neuroinflammatory environment through chemokine and cytokine receptors that they express, and actively participate in modulating the inflammatory environment by secreting chemokines and cytokines (Watson et al., 2020, 2021; Belenguer et al., 2021; Shariq et al., 2021; Willis et al., 2022; Dittmann et al., 2023). Finally, an elegant study by Marteyn et al. (2016) found that transplanting human NPCs, but not human OPCs, into the brain of Pelizaeus-Merzbacher (hypomyelinating leukodystrophy) mouse model leads to increased survival rates. While transplanted OPCs were found to myelinate the brain faster, NPCs were myelinating the brain slower, but were the only transplanted progenitors that successfully attenuated neuroinflammation (Marteyn et al., 2016). Specifically, transplanted OPCs maintained inflammatory (M1-like) status of microglia, which is associated with secretion of pro-inflammatory cytokines, whereas NPCs induced a shift toward repair state (M2-like) microglia, which are associated with secretion of anti-inflammatory cytokines (Marteyn et al., 2016). Thus, while the most significant role of NPCs and OPCs is attributed to their regenerative (neuron and macroglia replacement) properties, recent reports provide evidence that CNS progenitors also have immunomodulatory functions. From these OPC/NPC-immune functions and interactions, of particular interest are microglia-progenitor interactions as an example of cell communication between long-lived self-renewing cells of different origin (neurepithelium vs. yolk sac). Moreover, microglia themselves regulate much of NPC/OPC biology and are thus an active focus of research (Su et al., 2014; Miron, 2017; Sirerol-Piquer et al., 2019; Pérez-Rodríguez et al., 2021; Revuelta et al., 2022). However, the reciprocal cell-cell interaction implicating the role of NPCs and OPCs in regulating microglia biology has been less explored. Notably, the intersection of OPCs and immune cell interaction has recently been summarized in an elegant review (Kirby and Castelo-Branco, 2020). This review will focus on a new and emerging question of how NPCs communicate with microglia and regulate their fates, such as survival, proliferation, migration, phagocytosis and activation. For the latter, we will provide details including the author-reported microglia markers, such as cytokines and chemokines. In addition, we will provide conclusions reached by authors with regard to the categorization of microglial activation toward inflammatory (formerly M1) or repair (formerly M2) states.

## Effect of transplanted NPCs on microglia fates

The first evidence supporting the hypothesis that NPCs may regulate neuroinflammation came from transplantation studies. While the main role of transplanted NPCs was believed to be cell replacement, studies demonstrate that these NPCs may exert beneficial effects on degenerating and/or injured CNS primarily *via* trophic and immunomodulatory functions (Martino et al., 2011; Kokaia et al., 2012; Marteyn et al., 2016).

**FIGURE 1:**
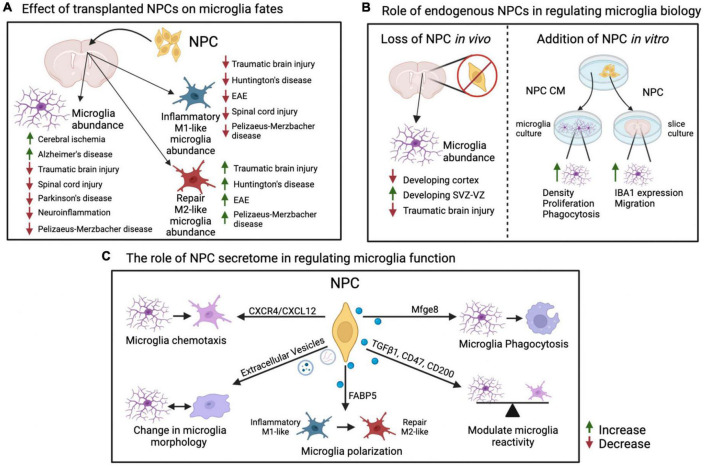
Summary of effects of NPCs on microglia fates. **(A)** Effects of transplantation of exogenous NPCs on microglial abundance and reactive states in various inflammation and disease models. Increase in total or M1-like/M2-like microglia abundance within a model is indicated with an upward green arrow, decrease is indicated with a downward red arrow. **(B)** Endogenous role of NPCs on microglia activity and localization *via* ablation of NPCs *in vivo* or addition of NPCs *in vitro*. **(C)** Factors and pathways originating in NPCs that have specific effects on microglial reactivity and functions. Please see text for details and references. This figure was generated using BioRender and Adobe Illustrator. NPC, neural precursor cell; EAE, experimental autoimmune encephalomyelitis; CM, conditioned media; SVZ, subventricular zone; VZ, ventricular zone.

Neurosphere cells derived from rodent SVZ or human fetal NPCs promote neuroprotection and recovery in rodent and human primate models of EAE when transplanted intravenously or intrathecally (Pluchino et al., 2003, 2005, 2009). Intravenously injected NPCs localize in inflamed CNS perivascular areas in close proximity to inflammatory CD45^+^ immune cells. These NPCs retain undifferentiated state and express α4 subunit of the integrin very late antigen (VLA)-4, which is usually associated with immune cells (Pluchino et al., 2005). Moreover, these NPCs spontaneously adhere to VCAM-1 (vascular cell adhesion molecule 1) expressing cells similarly to mitogen-activated CD4^+^ cells. This may explain specific recruitment of transplanted NPCs to CNS vasculature. Finally, NPCs express several receptors for inflammatory cytokines and chemokines, such as CCR1, CCR2, CCR5, CXCR3, and CXCR4 (Pluchino et al., 2005; Watson et al., 2020), which poises NPCs to respond to inflammatory environment. The positive effect of transplanted NPCs on EAE disease course and severity is believed to be at least in part due to NPC-mediated (i) increased numbers of reactive microglia capable of producing pro-apoptotic signals (Pluchino et al., 2005); (ii) increased cell death of CNS-infiltrating encephalitogenic T-cells (Pluchino et al., 2005); and (iii) decreased infiltration of CD45 + mononuclear phagocytes (Peruzzotti-Jametti et al., 2018; Figure 1).

Additional studies show that injection of NPCs leads to changes in microglia density and/or switch between inflammatory (formerly M1) and repair state associated with anti-inflammatory cytokine secretion (formerly M2) (Figure 1; Kokaia et al., 2012; Giusto et al., 2014; Zhou et al., 2022).

In support of the former, intracerebral infusion of mouse NPCs into mice with induced cerebral ischemia, and not healthy mice, increases density of CD11b^+^ cells in striatum (Capone et al., 2007). This is corroborated by another report, which demonstrates intracerebral transplantation of human NPCs into forebrain of neonatal rat pups with hypoxic–ischemic (HI) brain injury leads to increased density of striatal, but not cortical, IBA1^+^ cells (Daadi et al., 2010). Furthermore, intracranial transplantation of human NPCs increases number of microglia in a mouse model of Alzheimer’s disease (AD) (McGinley et al., 2018). In contrast, human or mouse NPC transplantation into the CNS of mouse models of traumatic brain (TBI), SCI, prenatal white matter injury (WMI), or Parkinson’s disease (PD) decreases microglia and/or macrophage density (Cusimano et al., 2012; Zuo et al., 2015; Gao et al., 2016; Koutsoudaki et al., 2016; Borhani-Haghighi et al., 2019). The difference in NPC-mediated action on total microglia/macrophage density could be attributed to the nature of CNS injury and/or the specific reactive status of microglia/macrophages that transplanted NPCs may be affecting.

In support of the latter, transplantation of human fetal NPCs into an AD mouse model leads to decrease in expression of major pro-inflammatory mediators, like IL-1β, IL-6, TNF-α, and iNOS (inducible nitric oxide synthase) (Lee et al., 2015). Injection of NPCs induced from mouse fibroblasts (iNPCs) into the murine brain with TBI reduces the density of inflammatory (M1-like) microglia (TNF-α^+^/IBA1^+^) and increases the density of IGF1^+^ (insulin growth factor 1) microglia, which are associated with trophic neuroprotective effect (Gao et al., 2017). Additional reports demonstrate transplanted NPCs in a mouse model of TBI show decrease in myeloid cells expressing CD86 and CD16/32, which are associated with pro-inflammatory state, and an increase in myeloid cells expressing CD206 and Arg1 (arginase 1), which are associated with repair or anti-inflammatory state (Gao et al., 2016). In agreement, transplantation of human neural stem cell line CTX0E03, but not fibroblasts, into quinolinic acid-lesioned rat striatum (model of Huntington’s disease) decreased inflammatory (M1-like) iNOS^+^ and increased repair (M2-like) CD206^+^ microglia/macrophages (Yoon et al., 2020). Rodents with SCI followed by NPC transplantation display (i) a drastic decrease in inflammatory M1-like macrophages, but not a significant change in repair M2-like macrophages; (ii) an increased proportion of ramified microglia; and (iii) reduced expression of P2X4, a purinergic receptor known to be upregulated in reactive microglia (Ulmann et al., 2008; Cusimano et al., 2012; Martínez-Rojas et al., 2022). Finally, transplantation of a cocktail of NPC-like cells derived from bone marrow and engineered to express molecules involved in inflammatory response modulation (NT-3 [neurotrophin-3], IL-10 and LINGO-1-Fc [Leucine rich repeat and Immunoglobin-like domain-containing protein 1]) suppresses murine EAE at least in part *via* decreasing iNOS^+^ inflammatory M1-like microglia and increasing Arg1^+^ repair M2-like microglia (Li et al., 2016). Thus, transplanted NPCs may tip the balance between inflammatory and repair microglia/macrophages leading to a change in neuroinflammatory environment and beneficial recovery from CNS injury (Figure 1).

## Role of endogenous NPCs in regulating microglia biology

With regard to endogenous NPCs, Brousse et al. (2021) have shown that the density of reactive microglia in the demyelinated corpus callosum of 2–4-month old mice is inversely proportional to the SVZ NPC density. While ablation of SVZ NPCs *via* ganciclovir infusion in healthy adult mice, which express thymidine kinase (TK) under the control of the NPC-specific Nestin promoter, does not lead to changes in microglia numbers (Butti et al., 2012), ablation of SVZ NPCs under pathological conditions, such as with TBI, results in fewer IBA1^+^ microglia and/or macrophages at the injury site (Dixon et al., 2015). In the developing brain, embryonic cortices with reduced basal progenitors achieved *via* constitutive knockouts of Emx2 or Pax6, or conditional Tbr2 knockout in NPCs, display a concomitant decrease in microglial cell numbers (Arnò et al., 2014). However, ganciclovir-mediated death of NPCs electroporated with TK expression vector leads to clustering and increased proliferation of microglia in the VZ-SVZ areas of the developing cortex (Arnò et al., 2014). In contrast, ganciclovir-mediated ablation of DCX^+^ neuroblasts, leads to abolishment of neurogenesis in SGZ and SVZ without any gross effect on the number of OX42^+^ microglia (Jin et al., 2010). Thus, it is possible that NPCs, and not neuroblasts, engage in cellular communication with microglia in neurogenic niches.

While often microglia and macrophages could not be distinguished in reports cited above, several elegant reports utilized co-culture of NPCs with isolated microglia or brain slices lacking macrophages. Wu et al. (2014) demonstrate NPCs co-cultured with murine adult brain slices in transwells increase expression of IBA1 and microglia migration. Media conditioned by murine neonatal cortical NPCs increase bead phagocytosis, density and proliferation of primary microglia and BV2 microglia-like cell line (Mosher et al., 2012). Interestingly, the secretome of microglia exposed to media conditioned by NPCs does not completely switch between inflammatory and repair profiles. Instead, NPC conditioned media causes multiple changes in secretion of both pro- and anti-inflammatory cytokines by primary microglia (Mosher et al., 2012). Finally, a cocktail of NPCs expressing NT-3, IL-10, and LINGO-1-Fc described above decreases expression levels of pro-inflammatory IL-1β, Nos2 (nitric oxide synthase 2) and TNF-α in transwell co-cultures with microglia and astrocytes (Li et al., 2016). These studies demonstrate NPCs directly affect microglial function *in vitro* through secretion of signaling molecules. Do NPC secreted molecules also have a similar effect *in vivo*? Mosher et al. (2012) demonstrate injection of NPCs or media conditioned by NPCs into the striatum of 2-month-old mice increases density, reactivity and proliferation of microglia (Figure 1).

## The role of NPC secretome in regulating microglia function

What are the molecules that can be mediating these effects on microglia? During development, basal progenitors located in the embryonic cortical SVZ express CXCL12 (C-X-C motif chemokine ligand 12) to recruit microglia, which express CXCR4 (C-X-C chemokine receptor type 4), an obligate receptor for CXCL12 (Arnò et al., 2014). Overexpression of CXCL12 in radial glia and basal progenitors *via in utero* electroporation leads to increased microglial numbers in the VZ-SVZ. Conversely, pharmacological block of CXCL12/CXCR4 signaling through injection of AMD3100, a pharmacological inhibitor of CXCR4 signaling axis, or constitutive knockout of CXCL12 in radial glia and basal progenitors (Gfap^Cre^:Cxcl12^flox/flox^) reduces the numbers of cortical microglia (Arnò et al., 2014). *In vitro* experiments demonstrate CXCL12 primarily increases microglial chemotaxis (Arnò et al., 2014). In contrast, radial glia located in the VZ of embryonic cortex express MIF (macrophage migration inhibitory factor), which promotes microglia proliferation *via* CD74 receptor (Arnò et al., 2014). Together, these results highlight the importance of embryonic NPCs for microglial migration, proliferation and positioning into the germinal niches of the developing cortex.

The mechanism of NPC-mediated activation of microglia varies in the literature. McGinley et al. (2018) demonstrate that while intracranial transplantation of human NPCs into 12-week old mice that express amyloid precursor protein a mutant human presenilin 1 (mouse model of AD) increase the number of microglia that exhibit a reactive morphology, this effect is independent of pro-inflammatory mediators IL-1β and TNF-α. Gao et al. (2017) however, report that co-culture of mouse iNPCs with lipopolysaccharide (LPS) treated, but not basal, neonatal microglia in transwells results in reduced expression of the proinflammatory markers CD68, TNF-α, and phosphorylated p65 (Ser276) with a concomitant increase in the expression of IGF1 when compared to microglia cultured without iNPCs. Notably, the iNPCs, but not microglia, show elevated CXCR4 in co-culture conditions (Gao et al., 2017). This is not surprising since NPCs express many chemokine receptors (Watson et al., 2020). Mouse iNPCs pre-treated with AMD3100 fail to reduce the expression of pro-inflammatory markers in co-cultured microglia when compared to normal iNPCs. Finally, intracerebral injection of mouse iNPCs with CXCR4 knockdown into adult mice with closed head injury leads to increased numbers of total microglia and lower ratio of IGF1^+^ microglia when compared to control iNPC group (Gao et al., 2017). Thus, CXCR4 signaling axis in NPCs contributes to NPC-mediated microglial reactivity.

Brousse et al. (2021) show a subset of SVZ NPCs, which remain undifferentiated in the corpus callosum of adult cuprizone-demyelinated mice, express Mfge8 (milk fat globule-EGF factor eight protein), a known ligand that can modulate microglial function *via* Itgb3 (integrin subunit beta 3) receptor. They further elegantly demonstrate media conditioned by SVZ NPCs isolated from control mice and grown as neurospheres increases *in vitro* microglial phagocytosis of myelin debris, which is brought to control levels in media conditioned by SVZ NPCs cultured from Mfge8 knockout mice. Finally, they show re-supplementation of Mfge8 knockout SVZ NPC conditioned media with exogenous Mfge8 rescues this phagocytic phenotype (Brousse et al., 2021). Together, the results suggest that undifferentiated SVZ NPCs in demyelinated corpus callosum modulate the biology and function of microglia *via* the Mfge8-Itgb3 ligand-receptor interaction (Brousse et al., 2021). Intriguingly, ablation of SVZ NPCs *via* ganciclovir infusion in mice challenged with cuprizone-induced demyelination leads to decreased neuroprotection and increased axonal loss without changes in overall numbers of microglia (Butti et al., 2019). Whether this neuroprotective effect of SVZ NPCs is due to Mfge8-mediated modulation of microglial phagocytosis, as opposed to density of microglia, remains to be addressed.

Morton et al. (2018) demonstrate neonatal SVZ NPCs secrete small extracellular vesicles (EVs) *in vitro* and *in vivo*, which are preferentially taken up by microglia. Intracerebroventricular injection of EVs purified from media conditioned by N2a cells (Neuro2a, a mouse neuroblastoma cell line that can differentiate into neurons) or primary NPCs into neonatal mice leads to morphological changes in microglia than engulf them. Namely, these microglia lose their processes and become more rounded, which is indicative of activated phagocytic status (Morton et al., 2018). While a variety of different proteins, lipids and miRNAs are packaged into EVs, the authors demonstrate Let7 miRNA mimics the morphological changes in engulfing microglia as observed with entire NPC EVs preparation. With regards to transcriptional changes induced in microglia, RNA-sequencing analysis of microglia incubated with NPC EVs reveals ∼1,700 genes change their expression levels. The most enriched and overrepresented genes fall into immune system processes and inflammatory responses categories. These genes included IL-1α, IL-1β, and IL-6 (Morton et al., 2018). In a rat model of SCI, EVs purified from embryonic spinal cord NPCs and injected into the tail vein show reduced number of CD68^+^ microglia/macrophages and reduced expression of pro-inflammatory cytokines IL-1β, IL-6, and TNF-α at the injury site. *In vitro* experiments demonstrate EVs derived from these NPCs can also reduce the production of nitric oxide (NO) by LPS-stimulated microglia (Rong et al., 2019). The differences in the effect of NPC EVs on density and activation status of microglia is most attributed to healthy vs. injured CNS, where microglia play varying homeostatic as well as damage- and regeneration-associated roles.

Lee et al. (2015) investigated the effect of NPCs on microglia function *in vitro* by using co-culture or transwell culture of LPS-activated BV2 microglia-like cell line with fetal human NPCs. In both cultures, presence of human NPCs or their conditioned media decreased expression of IL-1β, IL-6, TNF-α, and iNOS in LPS-activated microglia. The authors further showed human NPCs in these cultures expressed TGF-β1 (transforming growth factor beta-1), IL-4, IL-13, CX3CL1 (fractalkine), CD47, and CD200, which are known to have anti-inflammatory properties (Lee et al., 2015). Finally, co-culture of human NPCs with BV2 microglia-like cell line that have TGF-βR2, Sirpa (signal regulatory protein α, CD47 receptor) or CD200R1 receptors knocked down leads to a significant increase in the expression of microglial pro-inflammatory mediators, suggesting that NPCs may mediate microglia reactivity *via* TGFβ1, CD47, and/or CD200 (Lee et al., 2015).

Peruzzotti-Jametti et al. (2018) demonstrate transplantation of NPCs into EAE mice reduces the level of succinate in the cerebrospinal fluid (CSF) and subsequent infiltration of mononuclear phagocytes. Succinate is a metabolite that is usually accumulated in inflammatory mononuclear phagocytes and induces the expression of IL-1β *via* HIF-1α (Tannahill et al., 2013). To determine whether NPCs exhibited effect on mononuclear phagocyte activation *via* succinate, LPS-treated macrophages were co-cultured with NPCs in transwells and their metabolic and transcriptional changes were recorded. LPS-treated macrophages exhibit reduced basal oxygen consumption rate (OCR) and increased extracellular acidification rate (ECAR) when compared to basal macrophages. However, LPS-treated macrophages co-cultured with NPCs show restoration of both OCR and ECAR values, which is indicative of their anti-inflammatory phenotype. Furthermore, LC-MS analysis of LPS-treated macrophages co-cultured with NPCs show reduction of extracellular and intracellular succinate. Finally, RNA-sequencing analysis demonstrates these changes are concomitant with decrease in expression of pro-inflammatory markers, such as IL-1β, Nos2, and TNF-α, and increase in expression of anti-inflammatory markers, such as Arg1 (Peruzzotti-Jametti et al., 2018).

Finally, Zhou et al. (2022) show application of media conditioned by NPCs, which were differentiated from human embryonic stem cells, to BV2 microglia-like cell line reduced pro-inflammatory mediators expression in a PPAR-γ (peroxisome proliferator-activated receptor gamma) dependent manner. The authors further show NPCs secrete FABP5 (fatty acid binding protein 5), which functions *via* PPAR-γ (Forootan et al., 2016; Zhou et al., 2022). While the authors did not demonstrate that FABP5 is responsible for NPC-mediated decrease in pro-inflammatory microglial activation, they did demonstrate that addition of recombinant FABP5 prevented LPS-mediated expression of pro-inflammatory iNOS, IL-1β, IL-6, and TNF-α in a concentration dependent manner (Zhou et al., 2022).

In summary, the above-mentioned reports suggest that NPCs regulate microglia function primarily *via* secreted proteins, such as chemokines and cytokines, as well as extracellular vesicles and metabolites (Figure 1).

## Conclusion

Reports discussed above indicate that NPCs exert immunomodulatory effect in the developing, injured, and degenerating CNS mainly *via* secreted factors. In the future, it would be valuable to determine the role of NPCs in modulating other immune cells that infiltrate aging or degenerating CNS. It will also be important to undertake a systematic analysis of all NPC secreted ligands (proteins, growth factors, miRNAs, etc.) and determine their effect on microglia function in a developing, healthy adult and injured brain. This information could be useful in both understanding the fundamental NPC-microglia communication and utilization of this information for potential therapies for degenerating or injured CNS that extends beyond regenerative (cell replacement) capacities of NPCs and capitalizes on their immunomodulatory properties. Moreover, it will also be important to consider the contribution of neuroinflammatory properties of fetal and adult NPCs to the pathology of neurodevelopmental disorders.

## Author contributions

MA and AV: conceptualization and writing – original draft. MA, KG, and AV: writing – editing. KG: figure creation. All authors contributed to the article and approved the submitted version.
